# The mechanism of action of digoxin requires the sodium-dependent inactivation of the sodium-calcium exchanger

**DOI:** 10.1126/sciadv.ady9596

**Published:** 2025-12-17

**Authors:** Kyle Scranton, Scott John, Marina Angelini, Rui Zhang, Andreas Schwingshackl, Joshua I. Goldhaber, Riccardo Olcese, Michela Ottolia

**Affiliations:** ^1^ Division of Molecular Medicine, Department of Anesthesiology and Perioperative Medicine, David Geffen School of Medicine, University of California, Los Angeles, Los Angeles, CA, USA.; ^2^ Division of Cardiology, Department of Medicine, David Geffen School of Medicine, University of California, Los Angeles, Los Angeles, CA, USA.; ^3^ Department of Cardiology, Cedars-Sinai Health Sciences University, Los Angeles, CA, USA.; ^4^ Department of Pediatrics, David Geffen School of Medicine, University of California, Los Angeles, Los Angeles, CA, USA.; ^5^ Department of Physiology, David Geffen School of Medicine, University of California, Los Angeles, Los Angeles, CA, USA.

## Abstract

For more than two centuries, digoxin has been used to treat heart failure by increasing the strength of cardiac contraction and, more recently, is used for heart rate control. The proposed, yet unproven, mechanism underlying digoxin’s positive inotropic effect is as follows: By inhibiting the Na^+^-K^+^ ATPase (NKA), digoxin partially dissipates the transmembrane Na^+^ gradient, which is used by the Na^+^-Ca^2+^ exchanger (NCX1) to extrude Ca^2+^ from myocytes, thus causing accumulation of cytosolic Ca^2+^ and therefore increased cardiac contractility. Here, we demonstrate that digoxin critically relies on a specific allosteric regulation of NCX1, known as Na^+^-dependent inactivation, to exert its positive inotropic effect, establishing the precise mechanism of action of this historic drug. These findings identify a distinct molecular target for the development of positive inotropes that avoid the undesirable effects associated with the blockade of NKA. As the structural information for the region involved with NCX1 Na^+^-dependent inhibition is well resolved, we provide the mechanistic foundation for drug development.

## Teaser

Digoxin enhances cardiac contractility through allosteric inhibition of NCX1 by cytosolic sodium.

## INTRODUCTION

Despite decades of substantial advancements in cardiovascular medicine, cardiovascular disease remains the leading cause of death globally, with heart failure alone affecting more than 64 million people worldwide (*1*). Modern-day approaches to the treatment of systolic heart failure focus on reducing the load on the heart (*2*, *3*). However, for some patients with advanced systolic dysfunction, positive inotropic agents remain an essential tool for disease management. While a variety of positive inotropes are commonly used, such as phosphodiesterase-3 inhibitors, β-adrenergic agonists, or calcium sensitizers, these drugs are costly, thus rarely available in underdeveloped countries. Moreover, they require intravenous administration and close cardiopulmonary monitoring, limiting their use mainly to acute settings (*4*).

In contrast, digoxin, a plant-derived cardiac glycoside that has been used for more than two centuries to treat heart failure, remains the only orally administered positive inotropic agent approved for long-term administration (*5*). Although its use has much declined in recent years due to its narrow therapeutic window and risk of toxicity, digoxin remains prescribed, used now primarily for rate control and heart failure with 1.5 million annual prescriptions in the United States (*6*) and continued global use due to its affordability and universal accessibility (*7*). In addition, the drug has been gaining interest for alternative uses, showing promises as an anticancer agent (*8*, *9*). Thus, understanding its mechanism of action could be beneficial to develop alternative, safer therapeutic agents.

Digoxin enhances contractility by raising intracellular Ca^2+^ in cardiomyocytes. While some reports suggest that digoxin directly activates the ryanodine receptor leading to extra Ca^2+^ release from the sarcoplasmic reticulum (SR) (*10*–*15*), its widely accepted primary role is to inhibit the Na^+^-K^+^ ATPase (NKA) (*16*–*19*). By blocking NKA, digoxin raises cytosolic Na^+^ levels, reducing the Na^+^ electrochemical gradient across the cell membrane. As the Na^+^-Ca^2+^ exchanger (NCX1) relies on this gradient to power Ca^2+^ extrusion, any reduction in this gradient will decrease NCX1 activity, resulting in increased cytosolic Ca^2+^ (*20*–*22*). However, whether the impaired NCX1 activity is directly due to alterations in Na^+^ gradient remains experimentally unexplored. This question is particularly relevant given that increased Na^+^ levels can also inhibit NCX1 function via a mechanism known as Na^+^-dependent inactivation, an allosteric regulatory process in which high intracellular Na^+^ induces a time-dependent decay in exchanger current (*23*–*27*). Given that digoxin-treated cells experience markedly elevated intracellular Na^+^ levels (*28*, *29*), we posed the question whether this allosteric inhibition of NCX1 contributes to the mechanism of action of cardiac glycosides.

To test this hypothesis, we investigated the effects of digoxin on a mouse line in which the native cardiac exchanger was modified via CRISPR-Cas9 to genetically remove the Na^+^-dependent regulation, as previously described (*27*). The single amino acid substitution, Lys^229^Gln (K229Q), was introduced in the native exchanger as it selectively eliminates the Na^+^-dependent inactivation without affecting any other NCX1 biophysical properties (*25*, *27*, *30*), including its response to changes in Na^+^ gradients. We found that in the absence of NCX1 Na^+^-dependent inactivation, digoxin failed to improve contractility in both isolated mouse hearts and live animals. Consistently, digoxin enhanced the Ca^2+^ transient amplitude in field-stimulated wild-type (WT) adult ventricular myocytes (*20*, *21*, *31*–*33*), but was unable to potentiate Ca^2+^ transients in K229Q ventricular myocytes.

Together, these results indicate that the allosteric inhibition of NCX1 by Na^+^-dependent inactivation is required for the inotropic effect of digoxin, revealing the mechanism of action of this class of therapeutic agents. By raising intracellular Na^+^ through the inhibition of NKA, digoxin engages this allosteric regulatory mechanism of NCX1, which reduces Ca^2+^ extrusion and leads to positive inotropic effect. These findings pave the way for an alternative class of inotropes that, by targeting the allosteric regulation of NCX1 by cytosolic Na^+^, can modulate cardiac inotropy.

## RESULTS

### Protein expression level of the NKA is unchanged in K229Q mice

To investigate whether the Na^+^-dependent inactivation of NCX1 is relevant for the action of cardiac glycosides, we assessed the effects of digoxin on mice expressing the exchanger mutant NCX1 K229Q. This substitution exclusively abolishes the regulation of NCX1 by intracellular Na^+^ (*25*, *30*) and animals carrying this mutation live to adulthood with no readily discernible behavioral or physical phenotypes, as previously described (*27*).

**Fig. 1.**
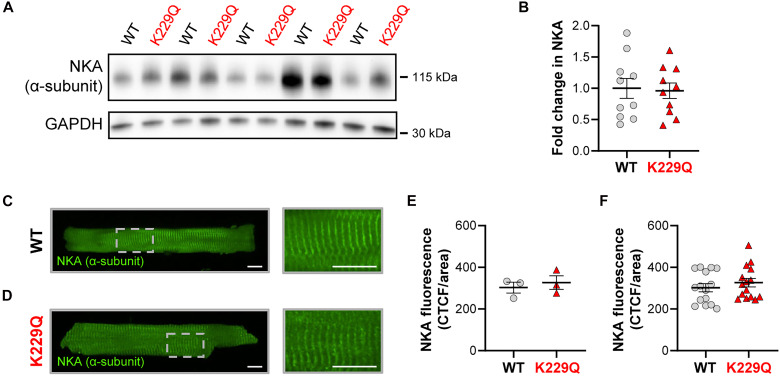
Mutation K229Q does not alter NKA protein expression levels in mouse heart homogenate. (**A**) Representative Western blot of NKA α-subunit (top) and GAPDH (bottom) expression of WT and K229Q ventricular homogenates. (**B**) Summary densitometry analysis showing the fold change in NKA protein expression between WT and K229Q mice, represented as NKA intensity/GAPDH intensity, normalized to WT levels. Hearts: WT, *n* = 10; K229Q, *n* = 10. Immunofluorescence image of WT (**C**) and K229Q (**D**) adult ventricular myocytes. Cells were stained with anti-NKA α-subunit antibody. The panel shows the magnified area of the cell. Cells in the image are maximum intensity projections. Scale bar, 10 μm. (**E**) NKA fluorescence is reported as corrected total cell fluorescence (CTCF) divided by cell area (CTCF/area). Each point represents one animal. Cells (four to six) were averaged for each animal. Animal averages were used for statistical comparisons. (**F**) Data from each individual cell, as in (E). NKA fluorescence was unchanged in K229Q myocytes compared to WT. Data are means ± SEM. Cells/animals: WT, *n* = 15/3; K229Q, *n* = 15/3.

As digoxin directly binds to and inhibits the NKA, we first determined whether removal of the NCX1 Na^+^-dependent inactivation resulted in altered NKA protein expression or trafficking. For this purpose, we conducted immunoblot analysis of ventricular homogenates isolated from WT and K229Q hearts. Our results show no significant difference in NKA α-subunit protein expression levels between hearts expressing the native exchanger and mutant K229Q (Fig. 1, A and B). In addition, immunostaining of adult ventricular myocytes indicates that NKA trafficking, distribution, and expression levels are not altered in K229Q myocytes (Fig. 1, C to E). This is consistent with a previous report that demonstrated no alterations in NKA α-subunit mRNA levels in K229Q mice (*27*). These findings suggest that the target substrate of digoxin is unchanged between the two models.

### Digoxin fails to generate positive inotropy in animals lacking Na^+^-dependent inactivation of NCX1

**Fig. 2.**
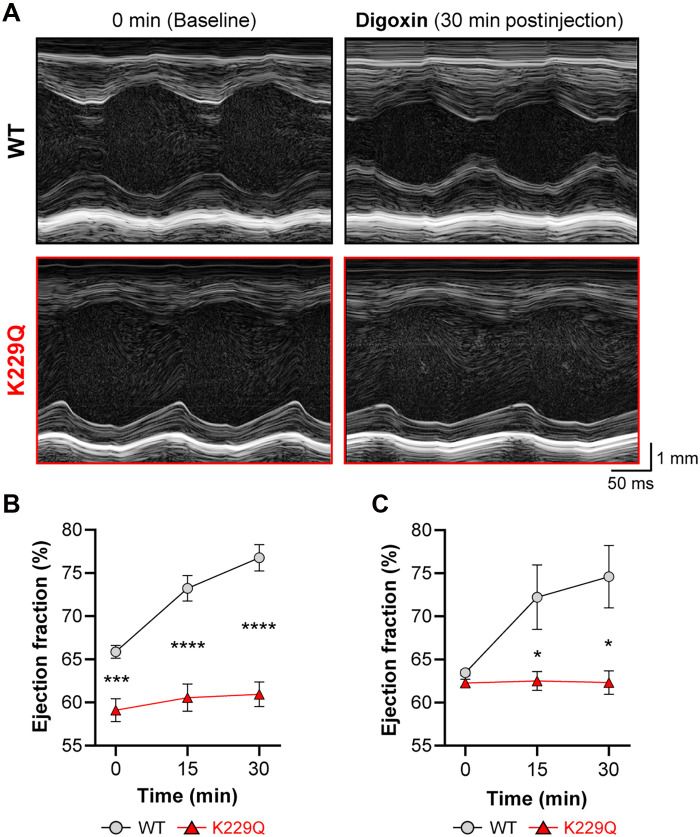
Digoxin-induced positive inotropy is abolished in K229Q mice lacking Na^+^-dependent inactivation. WT and K229Q mice were lightly anesthetized, and echocardiography measurements were taken before (baseline, time 0) and 15 and 30 min after digoxin (1 mg/kg) injection. (**A**) Shown are representative M-mode images of WT (top) and K229Q (bottom) hearts recorded at baseline (time 0) and 30 min postinjection of digoxin (right). (**B**) Summary plot of LV ejection fraction obtained at the indicated time after digoxin injection (WT, *n* = 10; K229Q, *n* = 10). (**C**) K229Q mice with comparable ejection fraction to WT failed to respond to digoxin administration, indicating that the lack of inotropic effect in K229Q mice is not related to their initial decreased contractility (WT, *n* = 4; K229Q, *n* = 5). Intraperitoneal injection of 1% DMSO in PBS alone had no effect on left ventricular ejection fraction or fractional shortening in both WT and K229Q mice (fig. S2). Data are means ± SEM. (*) indicates statistical comparison between WT and K229Q. ****P < 0.0001, ***P < 0.001, *P < 0.05.

Digoxin increases intracellular Na^+^ by inhibiting the NKA. The resulting reduction in the transmembrane Na^+^ gradient is widely considered the primary cause for NCX1 decreased Ca^2+^ extrusion (*34*, *35*). However, an alternative explanation may involve direct allosteric inhibition of NCX1 by elevated cytosolic Na^+^ levels via Na^+^-dependent inactivation. To test this possibility, we compared the effects of digoxin on cardiac function between control (expressing the native NCX1 exchanger) and K229Q mice, which lack Na^+^-dependent inactivation of NCX1 but remain sensitive to changes in the Na^+^ gradient. Echocardiography measurements were obtained either from lightly anesthetized (1.5 to 2.0% isoflurane) WT mice or K229Q mice and left ventricular function was recorded before (control, time 0) and every 5 min following intraperitoneal injection of digoxin (1 mg/kg), for a total of 30 min. As shown in Fig. 2A, WT mice showed a significant increase in ejection fraction (EF) within 15 min of digoxin administration, which further increased at the 30-min end time point (WT EF baseline = 65.87 ± 0.74%; 15 min of digoxin = 73.23 ± 1.49%; 30 min of digoxin = 76.78 ± 1.53%, *n* = 10 mice). As expected, fractional shortening (FS) was also significantly augmented after digoxin injection (WT FS baseline = 35.70 ± 0.55%; 15 min of digoxin = 41.73 ± 1.22%; 30 min of digoxin = 44.93 ± 1.31%, *n* = 10 mice). As intraperitoneal injection of vehicle alone had no effect on left ventricular EF or FS (fig. S1), these results demonstrate the ability of digoxin to enhance contractility in WT mice. In contrast, under identical experimental conditions, K229Q mice failed to respond to digoxin injection when investigated at the same time points, showing no significant changes in EF (Fig. 2B) (K229Q EF baseline = 59.11 ± 1.33%; 15 min of digoxin = 60.56 ± 1.58%; 30 min of digoxin = 60.95 ± 1.44%, *n* = 10 mice) or FS (K229Q FS baseline = 31.10 ± 0.85%; 15 min of digoxin = 32.01 ± 1.08%; 30 min of digoxin = 32.36 ± 1.01%, *n* = 10 mice).

As shown in Fig. 2B, the average baseline EF was reduced in K229Q mice compared to WT, a finding consistent with our previous report (*27*). To exclude the possibility that this decreased contractility at baseline underlies the insensitivity of K229Q mice to digoxin, we compared the effects of digoxin on WT and K229Q mice with similar initial EFs (Fig. 2C) (WT, *n* = 4; K229Q, *n* = 5). Figure 2C demonstrates that among these mice with comparable baseline cardiac functions, those carrying the Na^+^-insensitive exchanger, K229Q, showed no enhancement in EF.

To rule out that the aberrant response of K229Q mice to digoxin was elicited by changes in heart rate, we compared WT and K229Q heart rates throughout echocardiographic measurements. Figure S2 shows that both WT and K229Q live animals were similarly affected by digoxin’s negative chronotropic effect (*36*). This result indicates that the lack of K229Q mice response to digoxin is not due to altered heart rate and that the digoxin induced chronotropic effects are not driven by NCX1 Na^+^-dependent inactivation.

Together, these findings indicate that NCX1 allosteric regulation is indispensable for the positive inotropic effect of digoxin.

### NCX1 Na^+^-dependent inactivation is essential for the digoxin-induced enhancement of left ventricular developed pressure

We used the Langendorff isolated perfused heart assay to investigate the effects of digoxin perfusion on left ventricular developed pressure (LVDP). This ex vivo approach allows for studying the response of the heart to digoxin without the potential confounding effects of the autonomic nervous system (*36*) and other peripheral organ effects. This is particularly relevant given that the K229Q mutation is not limited to the heart but is present in all organs expressing the NCX1 isoform, including neurons (*37*).

**Fig. 3.**
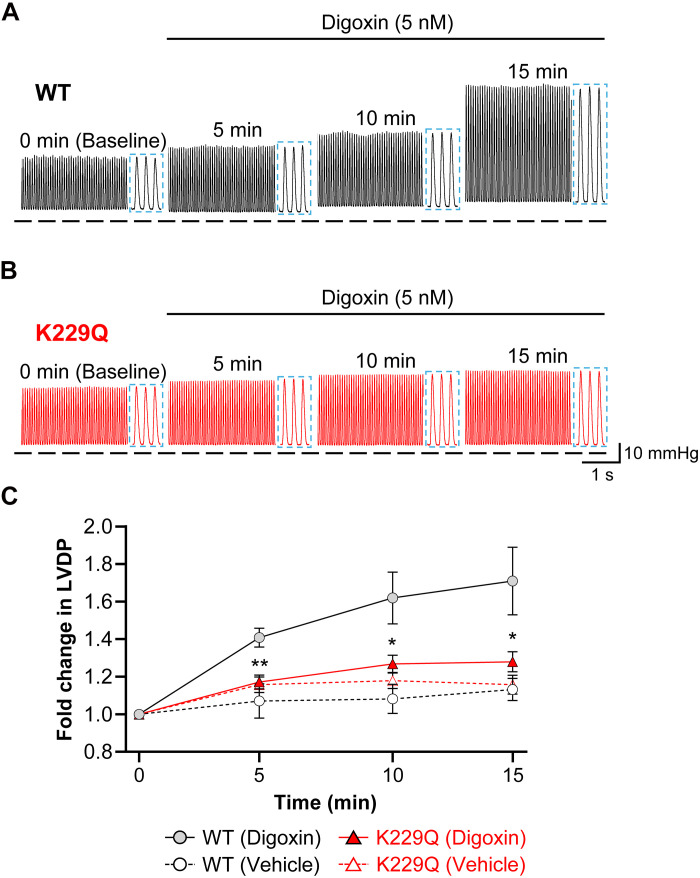
Digoxin fails to enhance contractility in K229Q ex vivo hearts. (**A** and **B**) Representative left ventricular developed pressure (LVDP) recordings from WT and K229Q Langendorff perfused isolated hearts. WT and K229Q hearts were excised and retrograde perfused at a constant pressure of 70 mmHg while externally paced at 6.25 Hz. LVDP was recorded via a pressure transducer placed in the left ventricle. After stabilization (baseline, time 0), hearts were perfused with digoxin (5 nM, 0.1% DMSO) or vehicle (0.1% DMSO) for 15 min. Dashed horizontal lines indicate 0 mmHg. Blue highlighted regions are displayed as dashed insets to the right of each time point recording. (**C**) Summary of the changes in LVDP normalized to baseline values in WT and K229Q hearts. Data are means ± SEM. Hearts: WT, *n* = 12; K229Q, *n* = 11. (*) indicates statistical comparison between WT and K229Q. **P < 0.01, *P < 0.05. LVPD significantly increased over time in WT hearts (*P* < 0.0001), whereas the modest changes observed in K229Q hearts were not significantly different from those in the presence of 0.1% DMSO (vehicle).

WT and K229Q mouse hearts were excised and cannulated onto a constant-pressure, gravity-fed Langendorff perfusion system and paced at 6.25 Hz via electrode stimulation of the apex of the heart. A pressure transducer (Millar) was placed into the left ventricle to record developed pressure. After 15 min of stabilization (baseline, time 0), a Tyrode’s solution containing 5 nM digoxin or vehicle [0.1% dimethyl sulfoxide (DMSO)] was perfused for 15 min while recording the LVDP. Examples of these recordings obtained at baseline (before digoxin) and after 5, 10, and 15 min of digoxin are shown in Fig. 3 (A and B). WT hearts showed a steady rise in LVDP, reaching ~60% increase at the end of the 15-min digoxin perfusion period. In contrast, K229Q hearts showed no statistically significant rise in LVDP when compared to the vehicle controls, indicating an impaired ability to elicit a complete inotropic response compared to WT mice. These results corroborate that the allosteric inhibition of NCX1 by cytosolic Na^+^ is necessary for the action of digoxin independently of any nerve input.

### NCX1 Na^+^ inhibition is required for digoxin-induced potentiation of the Ca^2+^ transient

To further understand the mechanism of action of digoxin, we examined its effect on Ca^2+^ cycling in both WT and K229Q isolated adult ventricular myocytes. Myocytes were loaded with the Ca^2+^-sensitive dye Fluo-4 AM and field stimulated at 1 Hz for 1 min to equilibrate the Ca^2+^ content in the sarcoplasmic reticulum (SR). Following this equilibration phase, control Ca^2+^ transients were recorded in the presence of 0.1% DMSO. Digoxin (5 nM dissolved in 0.1% DMSO) was then perfused for 5 min while cardiac myocytes were continuously paced at 1 Hz. Following the 5-min incubation time, Ca^2+^ transients were recorded from the same cells to compare their response.

**Fig. 4.**
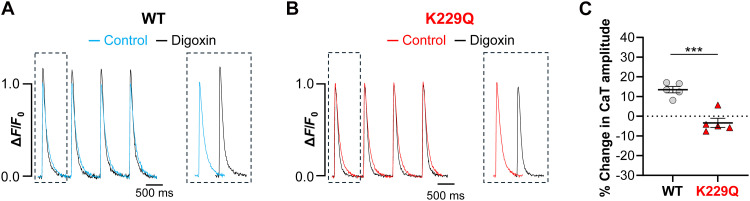
NCX1 Na^+^-dependent inactivation is required for digoxin-induced potentiation of the Ca^2+^ transient. (**A** and **B**) Examples of WT (A) and K229Q (B) field stimulated Ca^2+^ transients recorded before (WT: blue, K229Q: red) and after 5-min incubation with digoxin (5 nM, black traces). DMSO (0.1%) was present throughout the experiment, including during baseline recordings (control). Ca^2+^ transients were normalized to their respective baseline values (before digoxin, in the presence of 0.1% DMSO) and superimposed. Dashed insets show nonsuperimposed traces. (**C**) Graph summarizes the percent increase in Ca^2+^ transient (CaT) amplitude following digoxin application. Each point represents the average value for an individual animal, calculated from measurements in 17 to 36 cells. Data are means ± SEM. Animal averages were used for statistical comparisons. ***P < 0.001. The average obtained from single-cell measurements is shown in fig. S4.

Superimposition of normalized WT Ca^2+^ transients evoked before and after digoxin application (Fig. 4A) shows a significant enhancement of the Ca^2+^ transient amplitude in the presence of digoxin (Fig. 4, A and C). On average, the amplitude of WT Ca^2+^ transients increased by 13% in the presence of digoxin (WT = 13.52 ± 1.62%, *n* = 122/5 cells per mice). In contrast, K229Q Ca^2+^ transient amplitude failed to increase in the presence of digoxin, as shown in Fig. 4B and summarized in Fig. 4C (K229Q = −3.34 ± 2.34%, *n* = 149/5 cells per mice; *P* < 0.001).

The lack of response in K229Q myocytes is likely due to the mutation’s disruption of Na^+^-dependent inactivation. In the presence of digoxin, which raises intracellular Na^+^, the mutant exchanger remains active and continues to extrude Ca^2+^ efficiently. As a result, less Ca^2+^ is available for reuptake into the SR limiting SR Ca^2+^ loading and blunting digoxin’s positive inotropic effect. This interpretation is supported by our previous findings that K229Q myocytes display faster Ca^2+^ transient decay due to the enhanced activity of the mutated exchanger, while showing no changes in either sarcoplasmic/endoplasmic reticulum Ca^2+^ ATPase (SERCA) activity or SR load (*27*). Consistently, K229Q myocytes accumulate less diastolic Ca^2+^ than WT cells when paced at higher frequencies (*27*), conditions known to elevate both intracellular Ca^2+^ and Na^+^ levels (*38*, *39*).

To further analyze the effects of digoxin on Ca^2+^ dynamics, we determined whether removal of NCX1 Na^+^-dependent inactivation affects resting Ca^2+^ levels in the presence or absence of digoxin. To accomplish this, we measured the baseline Ca^2+^ fluorescence (diastolic Ca^2+^) from WT and K229Q myocytes paced at a constant frequency of 1 Hz. As shown in fig. S3, K229Q myocytes exhibited a modest reduction in baseline fluorescence compared to WT cells under control conditions and following digoxin application. However, while this difference was statistically relevant at the single-cell level (fig. S3A), it did not reach significance when comparisons between animals were analyzed using nested statistics (fig. S3B). The results suggest that resting Ca^2+^ levels are not greatly different between WT and K229Q myocytes, either at baseline or after digoxin treatment. One explanation is that the combination of slow pacing (1 Hz) and low digoxin concentration (5 nM) allows WT myocytes to effectively sequester excess cytosolic Ca^2+^ into the SR, enhancing Ca^2+^ transients without causing cytosolic Ca^2+^ overload. In contrast, the noninactivating K229Q exchanger remains active despite elevated intracellular Na^+^, promoting continuous Ca^2+^ extrusion, thereby preventing SR Ca^2+^ loading and ultimately blunting the digoxin-induced increase in Ca^2+^ transient amplitude.

### NCX1 Na^+^-dependent inactivation is not required for the positive inotropic effect of isoproterenol

**Fig. 5.**
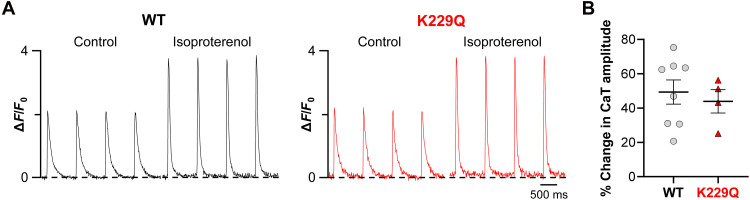
β-Adrenergic potentiation of Ca^2+^ transient amplitude does not involve NCX1 Na^+^-dependent inactivation. (**A**) Representative Ca^2+^ transients recorded from WT and K229Q myocytes before and after application of 1 μM isoproterenol. Cells were field stimulated at 1 Hz. Myocytes were incubated for 3 min with isoproterenol. (**B**) Summary data of the increase in CaT amplitude following treatment with isoproterenol. WT and K229Q showed similar responses, indicating that removal of NCX1 Na^+^ regulation does not alter the ability of myocytes to respond to agents that increase inotropy via Na^+^-independent mechanisms. Data are means ± SEM. Cells/animals: WT, *n* = 32/8; K229Q, *n* = 23/4. Cells (2 to 11) were averaged for each animal. Animal averages were used for statistical comparisons.

The experiments described above indicate that in the absence of Na^+^-dependent inactivation, K229Q cardiac myocytes are insensitive to the positive inotropic effects of digoxin. One possibility is that the enhanced Ca^2+^ extrusion by the K229Q exchanger, due to the absence of Na^+^ inhibition, prevents intracellular Ca^2+^ from rising sufficiently to fill the SR, preventing Ca^2+^ transient amplitude potentiation in the presence of digoxin. To test whether K229Q cardiac myocytes can increase intracellular Ca^2+^ sufficiently to enhance contractility via other known positive inotropic agents, we investigated their response to isoproterenol. Isoproterenol uses the β-adrenergic stimulation pathway to increase SR Ca^2+^ load with consequent potentiation of the Ca^2+^ transient amplitude, a pathway not reliant on changes in intracellular Na^+^. WT and K229Q myocytes were loaded with Fluo-4 AM and field stimulated at 1 Hz. Control Ca^2+^ transients were recorded after pacing the cells for 1 min to re-equilibrate SR load. Isoproterenol (1 μM) was added to the bath for 3 min while cells were continuously paced. Ca^2+^ transients were recorded from the same cells following the incubation period. Figure 5 compares the Ca^2+^ transient amplitude of WT and K229Q isolated ventricular myocytes before (control) and after 3-min incubation with isoproterenol (Fig. 5A). We found that WT and K229Q myocytes showed a similar increase in Ca^2+^ transient amplitude following isoproterenol incubation (Fig. 5B) (WT = 49.36 ± 7.06%, *n* = 32/8 cells per mice; K229Q = 43.96 ± 6.82%, *n* = 23/4 cells per mice). This indicates that myocytes lacking NCX1 Na^+^ inhibition can reach SR Ca^2+^ levels similar to WT mice through digoxin-independent mechanisms.

## DISCUSSION

Cardiac glycosides, such as digoxin, have been used for the treatment of cardiovascular disease for more than two centuries with their therapeutic use dating back to the late 1700s (*40*). They enhance contractility by raising intracellular Ca^2+^. The importance of NCX1 in mediating the inotropic effect of glycosides has been established as changes in its expression or activity drastically affect the effects of these drugs (*20*, *21*, *31*, *41*, *42*). However, the precise molecular mechanism underlying this relationship remains unclear.

**Fig. 6.**
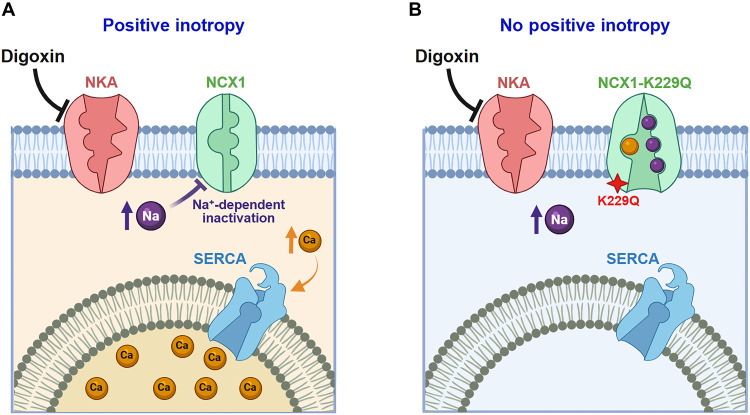
The positive inotropic effect of digoxin requires the Na^+^-dependent inactivation of NCX1. (**A**) Schematic representing the role of NCX1 Na^+^-dependent inactivation in the positive inotropic effect of digoxin. Digoxin binds to and inhibits the Na^+^-K^+^ ATPase (NKA), leading to an increase in intracellular Na^+^ levels. This elevation in Na^+^ in turn inactivates NCX1 through the Na^+^-dependent inactivation, thereby inhibiting Ca^2+^ efflux. The resulting rise in intracellular Ca^2+^ promotes SR Ca^2+^ loading, which underlies the positive inotropic effect of digoxin. (**B**) In the presence of the K229Q mutation, which disrupts Na^+^-dependent inactivation of NCX1, the digoxin-induced increase in cytosolic Na^+^ cannot inhibit exchanger activity. As a result, Ca^2+^ continues to be extruded from the cell, preventing SR Ca^2+^ accumulation and abolishing the positive inotropic response to digoxin.

The widely accepted mechanism of action of digoxin relays on its ability to selectively block NKA, resulting in the reduction of the Na^+^ gradient across the sarcolemma. As NCX1 uses the electrochemical gradient of Na^+^ to move Ca^2+^ out of the cell, the decreased driving force slows down its activity, leading to intracellular Ca^2+^ accumulation and thus positive inotropic effect (Fig. 6A). Our results challenge this traditional view by demonstrating that allosteric regulation of NCX1 by cytosolic Na^+^ (*24*, *27*, *37*, *43*, *44*) is essential for the inotropic action of cardiac glycosides. We show that digoxin increases intracellular Na^+^ by blocking NKA, which in turn inhibits NCX1 by triggering Na^+^-dependent inactivation without relying on Na^+^ gradient changes of the exchanger. This inhibition reduces Ca^2+^ extrusion, enhances SR Ca^2+^ content, and produces the positive inotropic effect (Fig. 6B).

Several key findings support this mechanism. First, the digoxin-induced positive inotropic effect is absent in both live K229Q mutant mice (Fig. 2) and isolated hearts (Fig. 3). Second, digoxin fails to increase Ca^2+^ transient amplitude in ventricular myocytes expressing the K229Q mutant exchanger (Fig. 4), consistent with the hypothesis that the K229Q exchanger continues to efficiently extrude Ca^2+^ in the presence of digoxin. We previously demonstrated that the K229Q mutant exhibits enhanced current under high intracellular Na^+^ conditions compared to WT NCX1 (*25*, *27*), supporting its capacity to maintain Ca^2+^ extrusion despite NKA inhibition. Last, we confirmed that neither the expression levels nor the cellular distribution of the NKA α-subunit are altered by the K229Q mutation (Fig. 1), effectively ruling out compensatory mechanisms. Similarly, the expression of other key proteins involved in excitation–contraction coupling remains unchanged in K229Q mice, as previously reported (*27*).

It is important to note that the removal of the Na^+^-dependent inactivation results in decreased cardiac contractility at baseline, as demonstrated in (Fig. 2), and as previously reported (*27*). We believe that this decreased cardiac function in K229Q mice at rest does not contribute to the altered response to cardiac glycosides. This is based on the observation that K229Q mice with an EF (62.28 ± 0.44%, *n* = 5 animals) similar to WT mice (EF: 63.45 ± 0.34%, *n* = 4) were insensitive to digoxin injection (Fig. 2C), while WT mice showed the expected positive inotropic response. The results indicate that despite comparable cardiac function, the animals expressing the mutant exchanger did not respond to digoxin administration, highlighting a direct role of the Na^+^-dependent inactivation in the action of cardiac glycosides. Moreover, myocytes expressing the mutant exchanger were able to raise intracellular Ca^2+^, and thus improve contractility, as efficiently as WT cells under β-adrenergic stimulation (Fig. 5). The result indicates that removal of NCX1 Na^+^-dependent inactivation does not impair Ca^2+^ accumulation in the SR in processes that are not dependent on cytosolic Na^+^ levels, further implicating the Na^+^-dependent inactivation as the ultimate target of cardiac glycosides.

In addition to enhancing cardiac contractility, digoxin also exerts a negative chronotropic effect, primarily through modulation of the vagal tone (*36*). In our mouse model, the K229Q mutant exchanger is expressed in all cells carrying the NCX1 isoform, including some neurons (*37*, *45*–*47*), raising the possibility that NCX1 regulation by cytosolic Na^+^ could contribute to this secondary effect of digoxin. However, our findings show that digoxin reduced heart rate to a similar extent in anesthetized WT and K229Q mice (fig. S2), suggesting that Na^+^-dependent regulation of NCX1 does not contribute to digoxin’s rate-lowering effect, but specifically affects its ability to enhance cardiac contractility.

Cardiac glycosides are known to affect organs other than the heart, including the kidneys, gastrointestinal tract, and central nervous system (*48*–*50*). Because some of these organs also express the mutant exchanger (*37*), they may contribute to the observed insensitivity of K229Q mice to digoxin. Our studies demonstrate that ex vivo hearts and isolated cardiac myocytes (which exclusively express the NCX1.1 isoform) (*51*) from K229Q mice did not respond to digoxin. These findings indicate that the presence of mutant NCX1 in cardiac muscle alone is sufficient to abolish the positive inotropic effect of digoxin, independent of contributions from other organs expressing the K229Q variant. Nevertheless, it remains possible that loss of Na^+^-dependent inactivation in peripheral organs could influence other physiological responses to digoxin that were not assessed here.

Last, some clinical studies have reported sex-based variation in the response and outcomes of digoxin therapy (*52*–*58*). Although these findings require further validation (*52*, *58*, *59*), sex differences in NCX and NKA expression, regulation, and subcellular localization in the heart may contribute to these differential responses (*60*–*67*). While inclusion of both sexes is essential for a comprehensive understanding of drug responses, these biological variabilities may confound interpretation of the specific role of Na^+^-dependent inactivation in digoxin’s effects. Therefore, in this study, we focused exclusively on male mice, enabling a more controlled investigation of the role of Na^+^-dependent inactivation in mediating the cardiac effects of digoxin.

The predominantly accepted mechanism of cardiac glycosides involves the inhibition of NKA activity, which in turn affects NCX1 Ca^2+^ extrusion capacity. However, other mechanisms of action have been proposed (*32*, *68*–*70*). Specifically, studies in cardiac SR vesicles, lipid bilayers, skeletal muscle vesicles, and isolated myocytes have reported direct activation or sensitization of the ryanodine receptor (RyR) by cardiac glycosides (*10*–*15*). While we did not directly investigate the effects of digoxin on RyR activity, our data show that removal of the exchanger Na^+^-dependent inactivation is enough to abolish the positive inotropic effects of digoxin. These findings indicate that cardiac glycosides exert their action mainly via a process driven by changes in intracellular Na^+^, as previously highlighted (*20*), implicating the allosteric inhibition of NCX1 as the main mediator of inotropy rather than a change in RyR activity.

Our findings support a paradigm shift in the understanding of the mechanism of action of cardiac glycosides: Their positive inotropic effect is mainly driven by the allosteric inhibition of NCX1 by intracellular Na^+^ and not the reduction of the Na^+^ electrochemical gradient. Hearts lacking this physiologically relevant regulation (K229Q) are not able to accumulate enough intracellular Ca^2+^ to gain a positive inotropic effect. Though digoxin remains as a prescribed drug, the well-defined toxicity and narrow therapeutic window due to the gross inhibition of the NKA has caused it to lose favor as a stand-alone inotrope (*71*). However, clinical studies continue to show promising signs for the use of cardiac glycosides in the management of heart failure (*72*). The revision to the canonical mechanism of action of cardiac glycosides described within this study paves the way for an alternative, safer class of positive inotropes highlighting the NCX1 Na^+^-dependent inactivation as a potential direct target.

## MATERIALS AND METHODS

### K229Q mouse line generation

The K229Q mouse line has been previously described (*27*). Briefly, mutation K229Q was inserted into the *Slc8a1* gene via CRISPR-Cas9. *Slc8a1* encodes for NCX1, including NCX1.1, the only exchanger expressed in the heart (*51*). The K229Q line was maintained in the C57BL/6J background. WT C57BL/6J mice were purchased from the Jackson Laboratory. This study used male 12- to 16-week homozygous K229Q mice throughout. The genotype was confirmed by Transnetyx using the following primers: forward: CAGCTCTCCTGGAGTTGTGG; reverse: TCCAAAACCAGAGCCCCATC.

Animals were housed in the University of California, Los Angeles (UCLA) Division of Laboratory Animal Medicine (DLAM) barrier facility under temperature/humidity-controlled conditions with a standard 12-hour light/dark cycle and were given free access to standard chow and water. Animal health and welfare was monitored by the UCLA DLAM veterinary care staff.

All animal protocols described herein were approved by the UCLA School of Medicine Animal Research Committee (ARC-2016-059) and strictly conformed to the *Guide for the Care and Use of Laboratory Animals* published by the United States National Institutes of Health.

### Echocardiography

Mice were lightly anesthetized with 1.5 to 2.0% vaporized isoflurane with supplemental oxygen and placed on a 37°C warming platform. B-mode and M-mode transthoracic echocardiography was performed using a VisualSonics Vevo 2100 with a 30-MHz linear transducer to analyze the cardiac hemodynamic parameters of WT and K229Q homozygous mice. Baseline echocardiographic images were taken for each mouse. Digoxin was given via intraperitoneal injection at the adjusted dosage of 1 mg/kg with the vehicle of 1% DMSO in phosphate-buffered saline (PBS). Echocardiographic images were recorded every 5 min following injection, up to 30 min. Mice remained anesthetized from the time of baseline recording to the termination of the experiment. Left ventricular EF and left ventricular FS were determined using parasternal short-axis M-mode images in Vevo LAB (5.5.1). Heart rate for each time point was determined by electrocardiography waves captured from paw electrodes during echocardiographic imaging.

### Langendorff isolated perfused heart

WT and K229Q mice were anesthetized with isoflurane. Hearts were excised and cannulated to a 21-gauge blunted needle attached to a constant-pressure (70 mmHg) Langendorff perfusion system. Hearts were perfused with a Tyrode’s solution (136 mM NaCl, 5.4 mM KCl, 10 mM Hepes, 1.8 mM CaCl_2_ 1.0 mM MgCl_2_, 0.33 mM NaH_2_PO_4_, and 10 mM glucose, pH 7.4) and bubbled with 100% O_2_. The left atrium was removed, and a pressure transducer (Millar, SPR-1000) connected to an amplifier (Bridge Amp, ADInstruments) was inserted into the left ventricle. LVDP was measured and recorded on LabChart Pro 8 (ADInstruments). Hearts were paced at the apex at 6.25 Hz with an in-house electrode attached to a stimulator (20 V, 8 ms square pulse; Grass S9D) and allowed to stabilize for 15 min. Subsequently, a digoxin (5 nM, 0.1% DMSO) or vehicle (0.1% DMSO) containing Tyrode’s solution was perfused for 15 min. All solutions and the heart were kept at 37°C with water-jacketed glassware and an inline solution heater (Warner Instruments).

### Isolation of adult ventricular cardiac myocytes

Ventricular cardiac myocytes were isolated from WT and K229Q mice. Mice were injected with heparin (200 UI/kg) to prevent blood coagulation, deeply anesthetized with 5% isoflurane (confirmed by abolished pain reflexes), and subjected to cervical dislocation before thoracotomy. Hearts were excised via aorta, pulmonary, and vena cava transection, and cannulated (21-gauge blunted needle) onto a gravity-fed, constant-pressure Langendorff perfusion system (Radnoti). Each heart was first perfused with Ca^2+^-free Tyrode’s solution (136 mM NaCl, 5.4 mM KCl, 10 mM Hepes, 1.0 mM MgCl_2_, 0.33 mM NaH_2_PO_4_, and 10 mM glucose, pH 7.4) for ~5 min, followed by perfusion of Ca^2+^-free Tyrode’s enzyme solution containing collagenase (type 2, 1 mg/ml, Worthington) and protease (type XIV, 0.1 mg/ml, Sigma-Aldrich) for 10 to 13 min. The enzyme solution was then washed out for ~8 min with Tyrode’s solution containing 0.1 mM CaCl_2_. All solutions were kept at 37°C with water-jacketed glassware. Ventricular tissue was excised and placed in a petri dish containing 0.1 mM CaCl_2_ Tyrode’s solution with bovine serum albumin (BSA) (10%) and gently shredded with forceps. Cardiac ventricular myocytes were separated from dead and contaminant cells via filtration through a 100 μM sterile, nylon mesh cell strainer (Fisher). Single cells were then reintroduced to 1.0 mM CaCl_2_ for 15 min and stored up to 5 hours in 1.8 mM CaCl_2_ at room temperature.

### Immunoblotting

Heart ventricular tissue (~50 mg) was isolated from WT and K229Q mice and homogenized on ice using Qiagen TissueRuptor in radioimmunoprecipitation assay buffer (500 μl) with a protease inhibitor cocktail (Roche cOmplete). Each homogenate was centrifuged at 16,000*g* for 20 min at 4°C. The supernatant was collected, and protein concentration was determined via a Pierce BCA assay kit (Thermo Fisher Scientific). Each lane represents a single heart homogenate with a total of 10 ventricles analyzed for each group. Each sample was treated with SDS/β-mercaptoethanol loading buffer and then loaded at a concentration of 15 μg of total protein per lane. Proteins were separated on 4 to 12% SDS–polyacrylamide gels (GenScript) and transferred to polyvinylidene difluoride membranes (Bio-Rad). The blot was divided to probe for all α-subunit isoforms of the NKA (a5, Developmental Studies Hybridoma Bank) and GAPDH as a loading control. Both sections were concomitantly blocked with 5% nonfat dry milk in PBS + 0.1% Tween-20 (PBST) for 1 hour at room temperature and probed with mouse monoclonal anti-NKA (1:5000; a5, Developmental Studies Hybridoma Bank, AB_2166869), or rabbit polyclonal anti-GAPDH (1:4000; 14C10, Cell Signaling Technology, cat. no. 2118) overnight at 4°C. Anti-mouse–horseradish peroxidase (HRP) (1:10,000; Sigma-Aldrich, cat. no. A3682) or anti-rabbit-HRP (1:10,000; Sigma-Aldrich, cat. no. A0545) was used to reveal protein bands via chemiluminescence (Immobilon Forte, Millipore). All antibody incubations were diluted in 5% nonfat dry milk in PBST. Images were captured with a Bio-Rad ChemiDoc XRS. Protein levels were quantified after blot background subtraction using Fiji ImageJ. Each lane of the NKA signal was normalized to its respective GAPDH signal. Uncropped scans of blots are in the Source Data file.

### Immunocytochemistry

Isolated WT and K229Q adult ventricular myocytes were placed on CellTak (Corning)–coated glass coverslips and fixed in 2% paraformaldehyde for 15 min at room temperature. Cells were blocked in PBS containing BSA (3%), goat serum (5%), and Triton X-100 (0.1%) for 1 hour at room temperature. Myocytes were then incubated with the mouse monoclonal anti-NKA α-subunit antibody (1:200, a5, DSHB) in 3% BSA PBS overnight at 4°C. Following primary antibody incubation, cells were washed 3 × 10 min in PBST (Sigma-Aldrich). Cells were then incubated with goat anti-mouse Alexa Fluor 488 (1:200, Abcam, cat. no. ab150113) for 1 hour at room temperature. Following secondary antibody incubation, cells were washed for 3 × 10 min in PBST and mounted (ProLong Gold, Invitrogen) to glass microscope slides. Immunofluorescence images were taken via laser-scanning confocal microscopy (Nikon A1) with a 40× objective. The same camera and laser settings were used for all WT and K229Q myocyte images. Cell images shown in Fig. 1 are maximum intensity projections. Corrected total cell fluorescence (CTCF) was measured using Fiji ImageJ (*73*) where CTCF = integrated density of cell − (area of cell × mean background fluorescence) (*74*).

### Ca^2+^ transients from isolated cardiac myocytes

Isolated WT and K229Q cardiac myocytes were incubated with 10 μM Fluo-4 AM (Invitrogen) and 0.1% Pluronic F-127 (Sigma-Aldrich) dissolved in Tyrode’s solution (1.8 mM CaCl_2_) for ~15 min at room temperature and mixed halfway through the incubation period. Cells were then washed with Tyrode’s solution and placed in a 35°C heated chamber with field stimulation (Warner Instruments). Before recordings, cells were given time to reach chamber temperature and then field stimulated via a 6-ms square pulse of constant voltage (10 V) using a MyoPacer Field Stimulator (IonOptix). Cells were imaged with an inverted microscope (Zeiss Axiovert 135) using an excitation filter (HQ480/40), an emission filter (HQ525/50), and a dichroic mirror (Q505lp) (Chroma). A 490-nm light-emitting diode (Thorlabs) was used as a light source. Images were acquired with a 10× lens using electron-multiplying charge-coupled device (EMCCD) cameras (Teledyne Princeton Instruments ProEM), operating at ~100 frames/s using a pixel count of 512 by 512 (binned to 256 by 256). LightField (Teledyne Princeton Instruments) was used to capture camera images.

To equilibrate the Ca^2+^ content in the SR, cells were paced at 1 Hz for 60 s before each recording. Following equilibration, 15 s “control” Ca^2+^ transient recordings were taken. To investigate the effects of digoxin, myocytes were placed in a chamber containing 0.1% DMSO from the start of the experiment, and this concentration was maintained throughout the protocol. After measurements of baseline traces, cells were treated with digoxin (5 nM, 0.1% DMSO), isoproterenol (1 μM), or vehicle (0.1% DMSO) during continuous pacing. All chemicals were added to Tyrode’s solution to the indicated final concentrations. Stocks of 5 μM digoxin and 1 mM isoproterenol were used, respectively. Ca^2+^ transient recordings were recorded every minute for 15 s following addition of the drug/chemical. The same cell was tracked during the experiment. After subtraction of nonspecific background fluorescence, the signal was measured from an identified region of interest within each single myocyte that encompassed ~80 to 90% of its total surface in the contracted state. Ca^2+^ transient amplitude was determined as (*F*_Peak_ – *F*_0_)/*F*_0_, abbreviated as Δ*F*/*F*_0_, where *F*_Peak_ and *F*_0_ are the peak and baseline fluorescence for each condition. Baseline fluorescence was reported as the resting fluorescence value between stimulation-induced Ca^2+^ release. Change in Ca^2+^ transient amplitude was determined as percentage change in amplitude calculated by subtracting the control Ca^2+^ transient from the posttreatment amplitude. For representation of digoxin Ca^2+^ transients (Fig. 4), traces were normalized to control amplitude and superimposed, as 0.1% DMSO itself causes a slight decrease in both Ca^2+^ transient amplitude overtime (fig. S5).

### Statistical analysis

All *P* values were calculated using an unpaired, two-tailed Welch’s *t* test. Normal sample distributions were assumed. *P* values are represented as follows: ****P < 0.0001; ***P < 0.001; **P < 0.01; *P < 0.05. To account for the nested structure of experiments involving individual cells (cells within animals), cell-level measurements were averaged per animal, and statistical comparisons were performed on these animal-level averages. *P* values are included in the figure legends. Statistical analyses were performed with Prism 9 (GraphPad). Analyses in which *P* > 0.05 are unmarked. Data are presented as means ± SEM, unless otherwise stated. Experimental “*n*” is noted throughout the text and distinguishes between the number of cells and animals used.
