# Supplementary material for: The mechanism of action of digoxin requires the sodium-dependent inactivation of the sodium-calcium exchanger
Source: Sci Adv. 2025 Dec 17;11(51):eady9596. doi: 10.1126/sciadv.ady9596 (PMC12710698; doi:10.1126/sciadv.ady9596)
Supplement: Supplementary file 1 — Figs. S1 to S5 [file sciadv.ady9596_sm.pdf]

Science Advances
AAAS

Supplementary Materials for

The mechanism of action of digoxin requires the sodium-dependent inactivation of the sodium-calcium exchanger

Kyle Scranton *et al.*

Corresponding author: Michela Ottolia, mottolia@ucla.edu

Sci. Adv. **11**, eady9596 (2025)
DOI: 10.1126/sciadv.ady9596

This PDF file includes:

Figs. S1 to S5

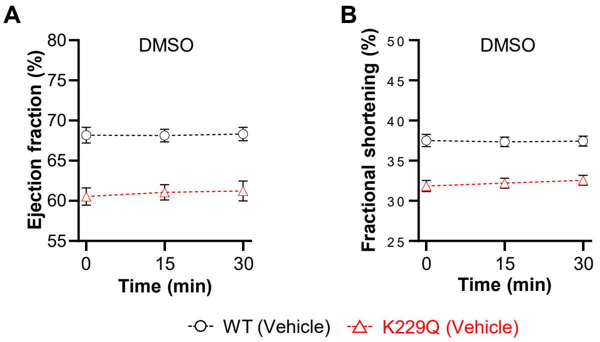

Figure S1. DMSO injection has no effect on ejection fraction or fractional shortening in WT or K229Q mice. A) Summary of ejection fraction of WT (black) and K229Q (red) mice measured at time 0, 15 minutes, and 30 minutes post-injection of 1% DMSO in PBS. **B**) Summary of fractional shortening of WT (black) and K229Q (red) mice measured at baseline, 15 minutes, and 30 minutes post-injection of 1% DMSO in PBS. Heart function was not statistically affected by DMSO injection in WT or K229Q mice. K229Q ejection fraction remained statistically lower than WT at all time points. Data are mean ± SEM. Animals: WT n = 8, K229Q n = 8.

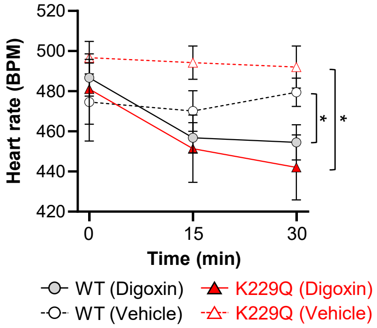

Figure S2. Digoxin-induced negative chronotropic effect does not require NCX1 Na^+ dependent inactivation. Heart rate of WT (grey/black) and K229Q (red) mice measured at time 0, 15 minutes, and 30 minutes post-injection of digoxin (1 mg/kg in 1% DMSO PBS) or vehicle (1% DMSO PBS). Both WT and K229Q mice exhibited a significant decrease in heart rate compared to vehicle-treated controls (1% DMSO in PBS), indicating that allosteric regulation of NCX1 by cytosolic Na^+ does not underline this effect of digoxin. Data are mean ± SEM. Animals: Digoxin WT n = 10, K229Q n = 10; Vehicle WT n = 8, K229Q n = 8. *P<0.05.

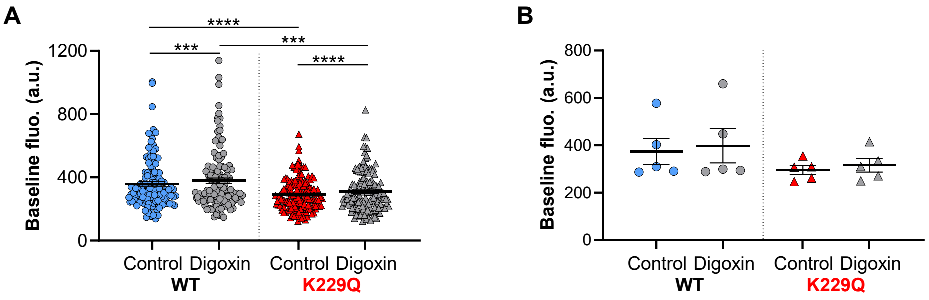

Figure S3. Resting levels of Ca^{2+} fluorescence measured from WT and K229Q myocytes. **A**) Graph summarizes the baseline (resting) Ca^{2+} fluorescence values of individual cells from WT and K229Q isolated ventricular myocytes before (control) and after 5-minute incubation with 10 µM digoxin. Cells were loaded in Fluo-4 AM. **B**) Average baseline Ca^{2+} fluorescence values for each animal (as in **A**). Although significant differences were observed at the individual cell level, these were not maintained when analyzed using nested statistics, indicating that the differences between animals were not sufficiently large or consistent. These results suggest that resting Ca^{2+} levels in WT and K229Q myocytes are not substantially different. Each point represents the average value for an individual animal, calculated from measurements in 17 to 36 cells. Data are mean ± SEM. Cells/animals: WT n = 122/5, K229Q n = 149/5. ***P<0.001, ****P<0.0001.

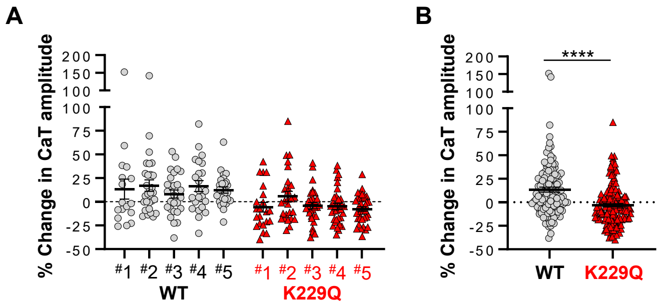

Figure S4. Na$^+$-dependent inactivation is required for digoxin-induced potentiation of the Ca^{2+} transient. A) Graph summarizes the percent increase in CaT amplitude following digoxin application for each individual WT (grey) and K229Q (red) cell within each animal used in Figure 3. Animals are indicated by numbering 1 to 5. **B**) Summary plot showing the average digoxin-induced Ca^{2+} transient potentiation measured from all WT and K229Q cells analyzed (WT n = 122, K229Q n = 149). Data are mean ± SEM.

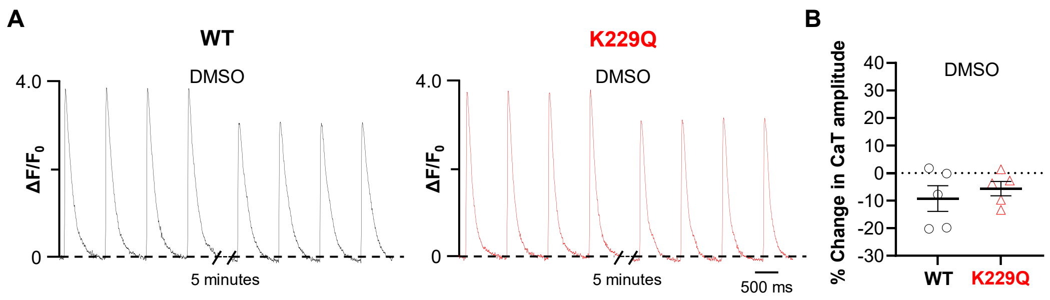

Figure S5. Perfusion of WT and K229Q myocytes with 0.1% DMSO decreases both baseline and peak fluorescence. A) Representative Ca^{2+} transients (CaT) of WT (black) and K229Q (red) ventricular myocytes recorded in the presence of 0.1% DMSO. After 5-minute perfusion with 0.1% DMSO in Tyrode's solution Ca^{2+} transients showed decreased amplitude. **B**) Percent change in CaT amplitude following 5-minute perfusion with 0.1% DMSO. . WT and K229Q myocytes showed a comparable reduction in Ca^{2+} transient amplitude following DMSO treatment, indicating that the absence of digoxin-induced Ca^{2+} transients potentiation in K229Q myocytes is not attributable to differential sensitivity to DMSO. Data are mean ± SEM. Cells/animals: WT n = 20/5, K229Q n = 27/5. Cells (2 to 5) were averaged for each animal. Animal averages were used for statistical comparisons.
